# Fungal Rhinosinusitis in Cats in the United Kingdom: 34 Cases (2013–2022)

**DOI:** 10.1111/jvim.70076

**Published:** 2025-03-28

**Authors:** Oliver Luke Russell, Daisy Johnson, Frederik Allan, Cameron Prior, Erin O'Connell, Perrine Henry, Daniel Thompson, Jack Fawsitt, Claudia Gil Morales, Victoria Neale, Irene Strelitzia Garcia Molina, Harriet Hall, Ruth Gostelow, Andre Kortum

**Affiliations:** ^1^ Queen's Veterinary School Hospital University of Cambridge Cambridge UK; ^2^ Vets Now Hospital Manchester Manchester UK; ^3^ Debenham Vets Surgical Referrals Stowmarket UK; ^4^ North Downs Specialist Referrals Bletchingley UK; ^5^ Royal Veterinary College University of London Hatfield UK; ^6^ Willows Veterinary Centre and Referral Service Referral Service Solihull UK; ^7^ Small Animal Teaching Hospital University of Liverpool Neston UK; ^8^ The Hospital for Small Animals University of Edinburgh Roslin UK; ^9^ Davies Veterinary Specialists Hitchin UK; ^10^ Langford Vets University of Bristol Langford UK; ^11^ Anderson‐Moores Veterinary Specialists Winchester UK; ^12^ Southfields Veterinary Specialists Basildon UK; ^13^ Dick White Referrals Six Mile Bottom UK

**Keywords:** aspergillus, cryptococcus, nasal, rhinitis, sinusitis

## Abstract

**Background:**

Fungal rhinosinusitis in cats is an uncommon condition with sparse literature regarding the presentation, causative agents, diagnosis, treatment, and prognosis within the UK.

**Hypothesis/Objectives:**

To describe and report the presenting clinical signs, diagnostic imaging findings, treatment approach, and outcome of cats diagnosed with fungal rhinosinusitis in the UK.

**Animals:**

Thirty‐four client‐owned cats were diagnosed with fungal rhinosinusitis.

**Methods:**

Retrospective multicenter observational study. Cases presenting at 10 UK referral centers between January 2013 and December 2022 were retrospectively recruited.

**Results:**

Median duration of clinical signs was 3 months (0.5‐42‐months). The most common signs were sneezing (27/34, 79%) and nasal discharge (21/34, 62%). Turbinate lysis was present in 27/34 cases (79%) and always involved the caudal nasal cavity. Osteolysis of the frontal bone, orbit, or cribriform plate was observed in 16/34 cases (47%). At least two tests from fungal culture, panfungal PCR, and histopathology were performed in all cases, and in 8/34 cases (24%) only one was positive. The treatment approach varied, with debridement, topical clotrimazole, and systemic azole therapy used alone or in various combinations, and repeat treatment occurred in 9/34 cases (26%). Clinical remission > 90 days after treatment was found in 9/24 cases (38%), but case fatality rates were low, with 3/34 cases (9%) dying with clinical disease during available follow‐up.

**Conclusions and Clinical Importance:**

Fungal rhinosinusitis should be considered in cats of any age with clinical signs of nasal disease. The prognosis from this data appears guarded for cats with fungal rhinosinusitis, with less than 50% of cats achieving long‐term clinical remission.

**Abbreviations**
CR: clinical remission
CT: computed tomography
FRS: fungal rhinosinusitis
MRI: magnetic resonance imaging
PCR: polymerase chain reaction

## 1 Introduction

Fungal rhinosinusitis (FRS) is an uncommonly reported condition in cats, and the literature is limited to small case series from variable geographical locations, apart from one larger Australian study of sinonasal and sino‐orbital aspergillosis in cats [1, 2, 3, 4, 5, 6, 7, 8, 9, 10, 11]. The most commonly reported causative agents of FRS in cats are *Aspergillus spp*. and *Cryptococcus spp*. However, other causative agents include *Penicillium spp*. [12], *Microsporum canis* [8], *Histoplasma capsulatum* [7], and *Scedosporium apiospermum* [13]. Information regarding the frequency and causative agents of FRS in cats within the United Kingdom is limited.

In contrast to dogs where dolichocephalic breeds are over‐represented [14, 15], brachycephalic cats might be predisposed to FRS caused by *Aspergillus spp*. [16] Although not proven, diabetes mellitus is considered a potential risk factor for sinonasal and sino‐orbital aspergillosis in cats [17]. Diagnosis of FRS in cats is challenging due to the level of operator skill required to achieve complete endoscopic visualization of the nasal and sinus cavities. Typically, diagnosis is based on cytological or histological identification of fungal hyphae or visualization of fungal plaques, alongside compatible clinical signs and diagnostic imaging findings [18]. Confirmation and characterization of the underlying causative organism rely upon fungal culture or panfungal PCR [17].

Due to the sparsity of reports and the variable underlying aetiologies, therapeutic recommendations are anecdotal or extrapolated from the treatment of sinonasal aspergillosis in dogs [17]. Treatment approaches described for FRS in cats caused by *Aspergillus spp*. (or less commonly *Penicillium spp*.) include a variable combination ofsystemic antifungal therapy with one or multiple of itraconazole, posaconazole, terbinafine, amphotericin B, and caspofungin; topical antifungal infusions; and debridement of fungal plaques [2, 5, 6, 10, 11]. However, there is no clear evidence available to guide the preferred therapeutic approach. Finally, prognostic information is limited for FRS in cats, particularly for cases caused by fungi other than *Aspergillus spp. or Cryptococcus spp*., but the prognosis is thought to be less favorable for more invasive and extensive presentations of aspergillosis [11, 17]. *Aspergillus spp*. associated with invasive FRS in cats, including *A. felis and A. udagawae*, can be phenotypically confused with *A. fumigatus*, which is typically associated with non‐invasive disease in cats, without molecular identification [11, 17, 19]. The former species can invade the nasal mucosa and progress to sino‐orbital aspergillosis, while the latter adheres to the mucosa and causes sinonasal aspergillosis [17, 19].

The aims of this study were to describe and report the presenting clinical signs, diagnostic imaging findings, treatment approach, and outcome of cats with FRS in the UK. Our hypotheses were that cats presenting with FRS would have a long history of nasal disease before presentation, with a chronic disease course characterized by a high incidence of treatment failure and relapse but low rates of case fatality relating to FRS.

## 2 Materials and Methods

A multicentre, retrospective observational study was undertaken involving 10 companion animal referral hospitals in the United Kingdom (Anderson Moores Veterinary Specialists, Davies Veterinary Specialists, Dick White Referrals, Langford Small Animal Hospital, Queen Mother Hospital for Animals, Queen's Veterinary School Hospital, Small Animal Teaching Hospital, Southfields Veterinary Specialists, The Hospital for Small Animals, Willows Veterinary Centre and Referral Service). Ethical approval was granted by (the Ethics and Welfare Committee of the Department of Veterinary Medicine, University of Cambridge (CR643)).

The medical record systems of each referral hospital were searched to identify cats diagnosed with fungalrhinitis, fungal sinusitis, or FRS between January 1st, 2013 and December 31st, 2022. A single investigator at each institution (VN, DT, HH, CGM, FA, JF, EO, IG, PH, CP, respectively) independently searched their respective database and assessed cases for eligibility. Due to variability in practice management software between institutions, search methodology was left to the discretion of each investigator. Cases were deemed eligible if FRS was considered the most likely diagnosis following investigations performed by a specialist‐led internal medicine service; advanced imaging (computed tomography (CT), or magnetic resonance imaging (MRI)) of the head had been performed; and one of the following was found during investigations to confirm a fungal etiology: Visualization of fungal plaques in the nasal cavity/frontal sinus; histopathological identification of fungal hyphae; positive fungal culture; or positive panfungal PCR. Cases were later excluded if they had suspected systemic fungal infections or if they were diagnosed with *cryptococcus spp*. rather than filamentous fungal causes of rhinosinusitis.

### 2.1 Data Collection

Data for eligible cases from each centre was collated by the named investigator into a single, anonymised spreadsheet (Microsoft Excel, Microsoft Corporation 2023) which was subsequently submitted to the primary investigator (OR).

Collected data relating to presentation included breed, age, sex, neuter status, presence of comorbidities, presenting clinical signs, duration of clinical signs before presentation, and whether the cat had any history of previous nasal disease. Further data was collected regarding diagnostic investigation, including diagnostic imaging, endoscopy, cytology, histopathology, fungal culture, and panfungal PCR. Finally, information was gathered regarding the treatment each case received (procedures performed, medications administered including dosage and dose frequency), and the outcome following treatment including whether clinical remission (CR—experiencing no sinonasal signs) was achieved, whether subsequent clinical relapses (relapse—recurrence of sinonasal signs) occurred, and survival time (number of days from first contact until final contact or death).

### 2.2 Statistical Analysis

Statistical analyses were performed using R version 4.3.0 for Mac (https://www.r‐project.org/) and Microsoft Excel (Microsoft Corporation 2023). Descriptive statistics were used to document findings, and non‐normally distributed data were reported as median with range, while normally distributed data were reported as mean with standard deviation. Categorical variables were reported as numbers and percentages. Continuous variables were assessed for normality using a frequency histogram and a Shapiro–Wilk test. Following initial data analysis, a further analysis of treatment and outcomes was performed for cases with FRS that were confirmed as caused by *Aspergillus spp*.

## 3 Results

### 3.1 Animals

Thirty‐nine cats eligible for enrollment were identified. Five were excluded: 1 due to a suspicion of systemic fungal disease; 2 due to histopathological evidence of nasal carcinoma; and 2 diagnosed with nasal cryptococcosis.

Thirty‐four cats were included in the final data analysis with a median age of 10.8 years (range 0.75–14.9 years) and a mean weight of 4.79 kg (S.D 1.16 kg). Apart from 1 cat that was 9 months old, all cats were > 5.75 years old. Of the 34 cats, 21/34 (62%) were castrated males and 13/34 (38%) were neutered females. Nineteen (19/34, 56%) were domestic shorthairs, with various other breeds also represented including 5/34 (15%) British shorthairs, 3/34 (9%) Persians, and 1/34 (3%) each of Bengal, Birman, British shorthair cross, Burmese, domestic longhair, Maine coon, and Siamese breeds. Brachycephalic breeds descended from Persian lineage (Persian, British shorthair) accounted for seven cases 7/34 (21%) and 12/34 (35%) were purebreds.

Systemic comorbidities were reported in 7/34 (21%) of cases. Chronic kidney disease (CKD) in 5/34 (15%) cats, 4 with IRIS stage II and 1 with IRIS stage III disease. Two cats (2/34, 6%) were hyperthyroid (receiving treatment), 1 with concurrent IRIS stage II CKD. Historically, 2/34 cats (6%) had tested positive for calicivirus. One cat (1/34, 3%) had diabetes mellitus, and 1 cat (3%) had pulmonary carcinoma. Retroviral testing was performed in 18/34 cats (53%) which tested negative for both FeLV and FIV. No cats had a known history of travel outside of the UK.

At the time of presentation, three cats were receiving immunosuppressive medication: Prednisolone 1.25 mg/kg per os q48 h in one cat, ciclosporin 7.4 mg/kg per os q48 h in one cat, and methylprednisolone 3.2 mg/kg by subcutaneous injection 2 months before presentation in one cat. These medications had been prescribed for long periods before presentation, and the reason for the prescription was not recorded in the clinical records.

### 3.2 Presenting Signs

The median duration of clinical signs before presentation was 3 months (range 0.5–42 months). Historical nasal signs that had resolved before the onset of more recent clinical signs were reported in 10/34 cats (29%). The most common presenting clinical signs were sneezing in 27/34 cats (79%), nasal discharge in 21/34 cats (62%) which was unilateral in 13/34 cases (38%) and bilateral in 8/34 cases (24%), stertor in 14/34 cats (41%), and epistaxis in 11/34 cats (32%). Coughing was noted in 8/34 cats (24%). Six cats (6/34, 18%) had ipsilateral local lymphadenomegaly. Four cats (4/34, 12%) exhibited exophthalmos, bilateral in 2/34 cases (6%) and unilateral in 2/34 cases. Additionally, 4/34 cats (12%) had paranasal swelling, 2/34 cats (6%) had pain on nasal palpation, 2 cats (6%) had a discharging sinus, 1 cat (3%) had nasal planum depigmentation, and 1 cat (3%) was reported to be pyrexic (rectal temperature > 39.2°C).

### 3.3 Diagnostic Imaging & Endoscopy

All cases had advanced imaging of the head performed; 32/34 cases (94%) underwent CT, and 2/34 cases (6%) underwent MRI. Turbinate lysis was documented in 27/34 cases (79%), bilateral in 13/27 cases (48%) and unilateral in 14/27 cases (52%). All 27 cases with turbinate destruction had changes present in the caudal nasal cavity, and 15/27 (56%) also had destruction of the rostral nasal turbinates. Disease involving the frontal sinus was reported in 21/34 cats (62%). Among the 7 cats that did not exhibit turbinate destruction, 2 had frontal sinus involvement.

Osteolysis of structures other than nasal turbinates was observed in 16/34 cases (47%). Of these: 3/16 (19%) had lysis of only the frontal bone; 3/16 (19%) had lysis of only the cribriform plate; 3/16 (19%) had lysis of the cribriform plate and orbit; 3/16 (19%) had lysis of only the orbit; 3/16 (19%) had lysis of the frontal bone and orbit; and 1/16 cases (7%) had lysis of the orbit, frontal bone, and cribriform plate. Of the cases with orbital lysis, 2 had orbital mass lesions. The 4 cases with exophthalmos noted on examination had no orbital masses reported on head CT.

Regional lymphadenomegaly was identified by advanced imaging in 15/34 cases (44%) of which only 6/15 cases (40%) had palpable lymph node enlargement noted on clinical examination. No cases had clinically identified lymphadenopathy that was not identified on advanced imaging of the head. Fungal rhinosinusitis was considered the most likely differential diagnosis based on the diagnostic imaging findings in 20/34 cases (59%).

Anterograde rhinoscopy was performed in 25/34 cases (74%) and turbinate destruction was directly visualized in 12/25 of these cases (48%). There were no cases where turbinate destruction identified at rhinoscopy had not been identified by advanced imaging. However, there were 9/27 cases (33%) with turbinate destruction identified by advanced imaging without this being evident endoscopically. Fungal plaques were identified by rhinoscopy in 15/25 cases (60%) where it was performed.

Based on a combination of diagnostic imaging and rhinoscopy findings, 27/34 cases (79%) were classified as having destructive rhinosinusitis. Of the remaining cases, 3/34 (9%) lacked destructive changes on imaging but had fungal plaques visualized on rhinoscopy or following frontal sinusotomy, and 4/34 cases (12%) had neither imaging evidence of destructive rhinosinusitis nor evident fungal plaques, but FRS was confirmed based on histopathology, microbiological testing, or molecular testing.

### 3.4 Fungal Culture, Panfungal PCR, Cytology, and Histopathology

Fungal culture was performed in 28/34 cases (82%) and was positive in 12/28 cases (43%). Panfungal PCR was performed in 11/34 cases (32%) and was positive in 8/11 cases (73%). The results of fungal culture and panfungal PCR are presented in Table 1. Both panfungal PCR and fungal culture were performed in 9/34 cases (27%) with panfungal PCR positive in 6/9 cases where culture was negative and culture positive in 2/9 cases where panfungal PCR was negative. There were zero cases where both tests gave positive results, and a total of 20/34 cases (59%) were positive on one of the two tests. Fungal species identified are displayed in Table 2. Two cases had two fungal species identified: 1 case was positive on fungal culture for both *Aspergillus fumigatus* and *Aspergillus versicolour*; 1 case was Panfungal PCR positive for both *Aspergillus spp*. and *Meyerozyma guilliermondii*. In this final case, as fungal hyphae were seen on nasal histopathology, *Meyerozyma guilliermondii* was likely a contaminant.

**TABLE 1:** Results of fungal culture, panfungal PCR and histopathological identification of fungal hyphae arranged by sample source.

	Fungal plaques		Nasal mucosal biopsies		Fungal plaque and mucosal biopsy		Nasal flush	
	Positive	Negative	Positive	Negative	Positive	Negative	Positive	Negative
Fungal culture (*n* = 28)	4	5	8	8	—	—	0	3
Panfungal PCR (*n* = 11)	3	1	4	2	1	0	—	—
Fungal hyphae on histopathology (*n* = 34)	7	1	12	2	12	0	—	—

*Note:* In some cases, samples were submitted for multiple tests, so the numbers of positive/negative results for a biopsy site might be greater than the number of cases that had biopsies collected from that site.

**TABLE 2:** Fungal speciation as identified fungal culture or panfungal PCR.

Fungal species	Fungal culture	Panfungal PCR
*Alternaria spp*.	0	1
*Aspergillus fumigatus*	10	3
*Aspergillus spp*.	1	3
*Aspergillus versicolour*	1	0
*Meyerozyma guilliermondii*	0	1
*Microascus spp*.	1	0
*Penicillium spp*.	0	1

*Note:* No samples were positive on both fungal culture and panfungal PCR. Therefore, the species identified all represent individual results with no agreement between tests. As fungal culture and panfungal PCR identified > 1 organism in some cases, there are more fungal species identified by culture/PCR than samples submitted for each test.

Cytology was performed on nasal biopsies in 9/34 cases (27%) and identified fungal hyphae in 6/9 cases (67%). All cases underwent histopathological examination, with fungal hyphae visualized in 31/34 (91%). Biopsies were taken fromfungal plaques in 8/34 cases (24%) with hyphae visualized in 7/8 cases; nasal mucosa in 14/34 cases (41%) with hyphae visualized in 12/14 cases (86%); and both locations in 12/34 cases (35%) with hyphae visualized in all 12/12 (100%; Table 2). It was not possible to identify which sample contained hyphae when both plaques and nasal mucosa were biopsied due to mixing of the samples before submission for histopathology. Fungal hyphae were identified histologically for all cases that had cytology performed.

When fungal hyphae were seen histologically (31 cases), panfungal PCR was positive in 7/31 cases (23%) and fungal culture was positive in 11/31 cases (35%). In 2 cases, fungal hyphae were not identified on histology, but panfungal PCR (*n* = 1) or fungal culture (*n* = 1) was positive. One case was histologically, panfungal PCR, and fungal culture negative and was diagnosed with FRS based on destructive rhinitis and visualization of fungal plaques by anterograde rhinoscopy.

### 3.5 Initial Treatment

Treatment was initiated before receiving histopathology, fungal culture, and panfungal PCR results in 7/34 cases (21%), and after receiving results in 27/34 cases (79%). Debridement of fungal plaques was performed in 18/34 cases (53%) and described as: Meticulous debridement of the nasal cavity and frontal sinus in 6/18 cases (33%); meticulous debridement of the nasal cavity only in 4/18 cases (22%); meticulous debridement of the frontal sinus only in 3/18 cases (17%); and partial debridement of the nasal cavity only in 5/18 cases (28%).

Topical antifungal therapy was applied in 11/34 cases (32%), administered into the nasal cavity through the nares in 4/11 cases (36%) and into the frontal sinus via sinusotomy in 7/11 cases (64%). The antifungal formulations used were: 1% clotrimazole solution in 7/11 cases (64%), 1% clotrimazole cream in 3/11 cases (27%), and 1% clotrimazole solution followed by 1% clotrimazole cream in 1/11 cases (9%). The duration of instillation of topical fungal medication was only known for 5 cases with a median time of 40 min (range 15–60). Nine (9/11, 82%) cases that had topical therapy administered had also undergone debridement of fungal plaques.

Systemic antifungals were administered to 26/34 cases (77%). The most common antifungal administered was itraconazole in 23/26 cases (89%), followed by fluconazole in 2/26 cases (8%), and posaconazole in 1/26 cases (4%). Itraconazole was administered once daily in 16/23 cases (70%) and twice daily in 7/23 cases (30%) at a median dose of 5 mg/kg/day (range 1.42–10). Fluconazole was administered twice daily in both cases at 4.0 or 17.2 mg/kg/day. Posaconazole was administered once daily at a dose of 7.5 mg/kg. Treatment with systemic antifungal therapy was recommended by the attending clinician for a median duration of 42 days (range 14–180).

**FIGURE 1:**
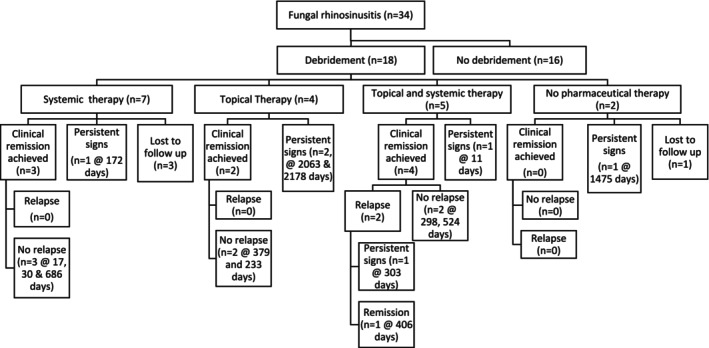
Treatment and clinical outcome flow chart for cases that underwent debridement. This flow chart illustrates the treatment options and outcomes for all cases that underwent debridement of their fungal disease. Additionally, the time of follow‐up is included for the outcomes.

**FIGURE 2:**
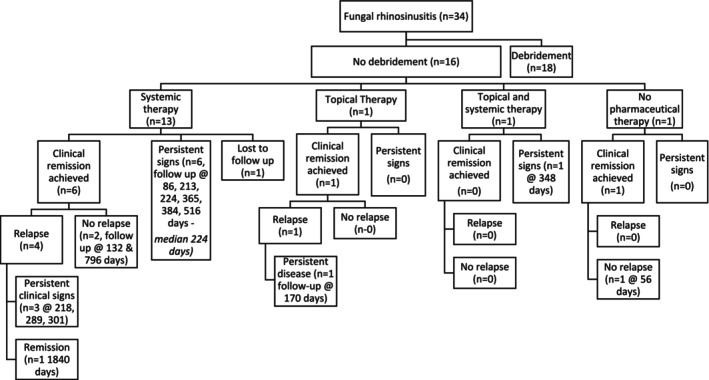
Treatment and clinical outcome flow chart for cases that did not undergo debridement. This flow chart illustrates the treatment options and outcomes for all cases that did not undergo debridement of their fungal disease. Additionally, the time of follow‐up is included for the outcomes.

More than one treatment was administered to 17/34 cases (50%) and included: Debridement and systemic treatment in 7/34 cases (21%); debridement, topical therapy, and systemic therapy in 5/34 cases (15%); debridement and topical therapy in 4/34 cases (12%); and topical therapy alongside systemic therapy in 1/34 cases (3%). Treatment options for all cases alongside outcomes are summarized in Figures 1 and 2.

Administration of topical therapy led to adverse effects in 2/11 cases, with 1 cat becoming hyporexic and another becoming head shy with serous nasal discharge. Adverse effects following systemic antifungal therapy occurred in 4/26 cases (15%), 2/2 receiving fluconazole and 2/23 receiving itraconazole (9%). With fluconazole, 1 cat developed hypersalivation on administration, prompting treatment discontinuation, and the other developed self‐limited diarrhea. One itraconazole‐treated cat developed self‐resolving vomiting, and another developed increased hepatobiliary parameters necessitating treatment discontinuation. Further information on this latter case was not available.

### 3.6 Clinical Outcomes

Outcome data was available for 29/34 cases (85%). Initial treatment led to the resolution of clinical signs in 19/29 cases (66%), improved clinical signs in 7/29 cases (24%) and no response in 3/29 cases (10%). In cases with the resolution of clinical signs, repeat investigations were performed at the time of initial reassessment in 3/19 cases (16%), comprising head CT in 2/19 cases (11%) and repeat rhinoscopy in another (1/19, 5%). One case (1/7) with improvement but not resolution of clinical signs underwent repeat head CT, which documented progressive disease.

Repeat investigations were performed in 9/34 cases (27%) at varying time points; in 4/9 cases because they never achieved CR, in 3/9 cases due to relapse, and in 2/9 cases to monitor response to treatment. Advanced imaging was repeated in 7 cases, with 6/34 cases (18%) undergoing CT and 1/34 (3%) MRI. Rhinoscopy was repeated in the 6/34 cases (18%) that had repeat advanced imaging, but none that did not. Fungal culture was repeated in 4/34 cases (12%). Repeat fungal culture was positive in 2/4 cases (50%) where it was performed. Panfungal PCR was repeated and negative in 1/34 cases (3%).

Further treatment beyond the initial treatment course was administered in 9/34 cases (26%); in 5/9 cases (56%) due to a failure to achieve CR and 4/9 cases due to relapse. Repeat treatment was administered topically only in 4/9 cases, systemically only in 4/9 cases, and both topically and systemically in 1/9 cases. Clotrimazole 1% solution was used in 3 cases and clotrimazole 1% cream in the other 2 cases. Posaconazole was used in 3 cases at a median dose of 7.5 mg/kg/day (range 5.6–8), with itraconazole 5 mg/kg/day and fluconazole 17.2 mg/kg/day used in 1 case each.

Clinical remission irrespective of repeated treatment was reported to occur in 17/34 cases (50%) of which 15/17 cases (88%) had an initial complete response to treatment, whilst 2/17 cases (12%) had a partial response to initial treatment. Cases entered CR a median of 77 days after initial presentation (range 17–479). Of the 17 cases that entered CR, 7/17 cases (41%) were reported to suffer relapse of clinical signs. The median time from CR to relapse was 106.5 days (range 32–220, data not available for 1 case). Treatment pathways, achievement of CR, and subsequent relapses for all cases are summarized in Figures 1 and 2.

Final follow‐up information was available at a median of 301 days (range 11–2178 days) after initial investigations for a total of 29 cases. Outcomes at the time of final contact are summarized in Table 3, and outcomes related to treatment are summarized in Figures 1 and 2. Follow‐up at > 90 days was available for 24 cases at a median time of 356.5 days (range 132–2178 days), and these results are summarized in Table 4.

**TABLE 3:** Clincial outcome data for 29 cases where available.

Clinical outcome	Remission achieved (*n* = 17)		Remission never achieved (*n* = 12)
	Relapse (*n* = 7)	No relapse (*n* = 10)	
In clinical remission (*n* = 9)	1 (3%)	8 (28%)	0
Died in clinical remission (*n* = 3)	1 (3%)	2 (7%)	0
Receiving ongoing treatment (*n* = 3)	1 (3%)	0	2 (7%)
Ongoing clinical signs (*n* = 11)	3 (10%)	0	8 (28%)
Died with ongoing clinical signs (*n* = 3)	1 (3%)	0	2 (7%)

*Note:* Reasons for death: Died in remission: One humanely euthanized due to pancreatic carcinoma, two unknown causes of death. Died clinically: Two humanely euthanized due to progressive fungal rhinosinusitis and one with an unknown cause of death but with progressive disease.

**TABLE 4:** Clinical outcome data for 24 cases where available at > 90‐days.

Clinical outcome	Remission achieved (*n* = 12)		Remission never achieved (*n* = 10)
	Relapse (*n* = 7)	No relapse (*n* = 7)	
In clinical remission (*n* = 6)	1 (4%)	5 (21%)	0
Died in clinical remission (*n* = 3)	1 (4%)	2 (8%)	0
Receiving ongoing treatment (*n* = 3)	1 (4%)	0	2 (8%)
Ongoing clinical signs (*n* = 9)	3 (13%)	0	6 (25%)
Died with ongoing clinical signs (*n* = 3)	1 (4%)	0	2 (8%)

*Note:* Reasons for death as per Table 3.

### 3.7 Treatment and Outcomes for Cases With Confirmed *Aspergillus Spp*.

Seventeen cases had confirmed *Aspergillus spp*. based on fungal culture or panfungal PCR. Debridement was performed in 10/17 cases (59%): Meticulous debridement of the nasal cavity and sinus in 4/10 cases (40%), partial debridement of the nasal cavity only in 3/10 cases (30%), meticulous debridement of the nasal cavity only in 2/10 cases (40%), and meticulous debridement of the sinus only in 1 case (10%). Topical therapy was administered in 8/17 cases (47%), 7/8 of which had undergone debridement: Clotrimazole 1% solution in 6/8 cases and clotrimazole 1% cream in 2/8 cases. Systemic antifungal therapy was administered to 11/17 cases (65%); itraconazole in 10/11 cases (91%) and posaconazole in 1/11 cases (9%). Three cases (3/17, 18%) received both topical and systemic therapy, 8/17 (47%) received only systemic therapy, 5/17 (29%) received only topical therapy, and 1 case (6%) received neither antifungal therapy nor underwent debridement.

Thirteen cases (13/17, 77%) entered CR a median of 77 days after initial presentation (range 17–351). Of the 13 cases that entered CR, 7/13 cases (54%) suffered a relapse. Median time from CR to relapse was 106.5 days (range 32–220, unavailable for 1 case). Final follow‐up data was available for all cases at a median of 298 days (range 17–2178) and are summarized in Table 5. Follow‐up data at > 90 days were available for 15/17 cases at a median of 301 days (range 170–2178), with these results displayed in Table 6.

**TABLE 5:** Clinical outcome data for 17 cases with confirmed *Aspergillosis spp*.

Clinical outcome	Remission achieved (*n* = 13)		Remission never achieved (*n* = 4)
	Relapse (*n* = 7)	No relapse (*n* = 6)	
In clinical remission (*n* = 5)	1 (6%)	4 (24%)	0
Died in clinical remission (*n* = 3)	1 (6%)	2 (12%)	0
Receiving ongoing treatment (*n* = 2)	1 (6%)	0	1 (6%)
Ongoing clinical signs (*n* = 5)	3 (18%)	0	2 (12%)
Died with ongoing clinical signs (*n* = 2)	1 (6%)	0	1 (6%)

*Note:* Reasons for death as per Table 3.

**TABLE 6:** Clinical outcome data for 15 cases with confirmed Aspergillosis *spp.* where available at > 90‐days.

Clinical outcome	Remission achieved (*n* = 13)		Remission never achieved (*n* = 4)
	Relapse (*n* = 7)	No relapse (*n* = 4)	
In clinical remission (*n* = 3)	1 (7%)	2 (13%)	0
Died in clinical remission (*n* = 3)	1 (7%)	2 (13%)	0
Receiving ongoing treatment (*n* = 2)	1 (7%)	0	1 (7%)
Ongoing clinical signs (*n* = 5)	3 (20%)	0	2 (13%)
Died with ongoing clinical signs (*n* = 2)	1 (7%)	0	1 (7%)

*Note:* Reasons for death as per Table 3.

### 3.8 Treatment and Outcomes for Phaeohyphomycosis

Two cases were diagnosed with phaeohyphomycosis. One with *Microascus spp*. received oral fluconazole at 17.2 mg/kg/day, never achieved CR, and was euthanized at 384 days due to the development of neurological signs presumed to be caused by the extension of fungal disease. One with *Alternaria spp*. received oral itraconazole at 4.93 mg/kg/day, never achieved CR, and was still alive with clinical signs at 365 days post‐presentation.

### 3.9 Cases With Concern for Orbital Lesions

Six cases (6/34, 18%) had concern for orbital lesions. Exophthalmos was reported on clinical examination in 4/34 cases (12%), but no mass lesions were reported on head CT, and in 2/34 cases (6%) there was no clinically reported exophthalmos but orbital mass lesions on head CT. Fungal culture identified *Aspergillus fumigatus* in 3/6 cases and was negative in the other 3 cases. Fungal plaques were visualized in the sinus in 3/6 cases and in the nasal cavity in 1/6 case. In all culture‐negative cases, fungal plaques were visualized.

Three cases (3/6) with concern for orbital lesions underwent frontal sinus debridement, and 1/6 of these cases received topical treatment with 1% clotrimazole solution followed by 1% clotrimazole cream. All 6 cases received systemic itraconazole at a median dose of 7.5 mg/kg/day (range 4–10). The 2 cases with orbital masses were treated no differently compared to the other 4 cases. Follow‐up was available for 5/6 cases: 2/5 cases were in CR at 132 and 686 days; 1 case was receiving ongoing treatment at 218 days; 1 died for an unknown reason with clinical signs at 289 days; and 1 had ongoing clinical signs at 11 days.

## 4 Discussion

This multicentre study reports the clinical signs, diagnostic findings, treatment approach, and outcomes for 34 cats diagnosed with FRS in the United Kingdom. Although breed prevalence in the respective hospital populations was not evaluated, a notable number of cats were brachycephalic (7/34 (21%) overall, 6/17 (35%) with confirmed aspergillosis) which is comparable to previous reports for aspergillosis in cats (33%) [17]. All brachycephalic cats were of Persian lineage, suggesting (as previously reported) that these cats might be genetically predisposed to FRS, as has been shown for dermatophytosis [17, 20]. In contrast to other studies which report a younger age demographic, all but two cats in our study were middle‐aged or older, with 19 cats exceeding 10 years of age [17]. This study therefore indicates that FRS should be considered as a differential diagnosis for all cats with chronic nasal disease regardless of breed or age.

The presenting clinical signs identified in this study were similar to those reported for cats suffering from FRS, with sneezing, stertor, and nasal discharge being the most common presenting signs [5, 17]. As hypothesized, some cats had a prolonged duration of clinical signs before referral, with 10/34 cats (29%) having a previous history of nasal disease. Although this warrants further investigation, it might suggest FRS can present with a more chronic disease course easily confused with other differential diagnoses such as idiopathic chronic rhinitis. Alternatively, underlying pre‐existing nasal disease might predispose individuals to the development of FRS.

Turbinate lysis was identified as being predominantly caudal in nature, which has not been reported. The finding of roughly equal numbers of cases affected unilaterally as bilaterally is contrary to the reported preponderance of bilateral disease [17, 18, 21]. Diagnostic imaging findings were otherwise similar to those reported for FRS in cats, with a high number of cases (16/34, 47%) displaying lysis of bony structures other than nasal turbinates, including the orbit, cribriform plate, and frontal bone [18, 21]. This frequency and extent of bony lysis are higher than reported in dogs, possibly suggesting a more aggressive disease process in cats, which might be due to the fungal organisms involved or a tendency for cats to be presented at a later stage of the disease [15]. Rhinoscopy appears useful for visualization of and debridement of fungal plaques in the nasal cavity, but in our study had a low sensitivity for identifying turbinate destruction compared to advanced imaging. Furthermore, considering the small size of the feline nasal cavity, endoscopic access to the ethmoid region or frontal sinus via anterograde rhinoscopy was not routinely possible; therefore, caudal nasal or sinus lesions might be missed without additional diagnostics such as advanced imaging or frontal sinusoscopy. However, the utility of rhinoscopy strongly correlates with operator experience, and in the hands of an experienced operator, it might be the single most valuable diagnostic test. Finally, three cases were diagnosed based on microbiological testing, molecular testing, or histopathology without evidence of destructive rhinosinusitis on CT/MRI or anterograde rhinoscopy. These cases reiterate the importance of further testing in cats with clinical signs of chronic nasal disease even in the absence of diagnostic imaging changes.

In this study, there was a lack of agreement between the various testing modalities (histopathology, panfungal PCR, fungal culture) used to confirm a diagnosis of FRS. In eight cases, only one diagnostic test (histological identification of fungal hyphae, panfungal PCR, fungal culture, or rhinoscopic visualization of fungal plaques) was positive. This highlights how extensive investigations might be required to confirm FRS in suspicious cases. Assessment of the sensitivities of various tests was limited by the lack of standard investigations, the variability in sample handling/processing, and the small sample size. Due to the retrospective, multicentre nature of this study, various sample types were submitted to various laboratories at multiple time points, meaning that fungal culture and panfungal PCR methodology were likely heterogeneous. Also, tissue type (fresh or formalin fixed) submitted for panfungal PCR and the type of panfungal PCR performed was not available for review.

In contrast to previous reports, topical antifungal therapy with both clotrimazole solution and clotrimazole cream was well tolerated in 4 cases in which it was used [22]. Itraconazole was the most common first‐line systemic antifungal, which likely relates to it being the only azole licensed for systemic administration in cats in the UK. Adverse effects were similar to those reported, and in general, systemic therapy was well tolerated, with 2 adverse events (2/26, 8%) necessitating treatment discontinuation [23]. Considering the small number of cases and the variability in treatment regimens, no firm recommendations regarding treatment can be made. However, with the relatively poor response rate, it seems reasonable to suggest that early, aggressive therapeutic interventions, including meticulous debridement, alongside investment in fungal speciation and antifungal sensitivity testing to guide appropriate therapy, are warranted to maximize the likelihood of CR and disease resolution.

The prognosis for FRS in cats, regardless of the causative agent in this study, was poor, with 12/29 cases (41%) being in CR at the time of final contact and 9/24 cases (38%) known to be in CR at > 90 days post‐initial presentation. However, at least 2 cases were suffering from phaeohyphomycosis, which might have a higher recurrence rate than FRS caused by *aspergillus spp*. [24, 25] Of cases with confirmed aspergillosis, 8/17 cases (47%) were in CR at the time of final contact, with 6/15 cases (40%) in remission at > 90 days post‐initial presentation. This is a poorer prognosis than that previously reported in the literature [11, 17] and might reflect the heterogeneity of the treatment approach or the challenges of confirming disease remission, given that CR might not be a reliable indicator of disease cure [26]. The limited number of cats undergoing repeat investigation to confirm true disease remission is a limitation but reflects the reality of managing these cases, where the costs of these procedures, especially following the extensive and costly diagnostic investigations and treatment interventions, are frequently prohibitive. Additionally, the 10 cases with no longer‐term follow‐up might have artificially reduced the reported treatment efficacy, as owners might be less likely to present their cat for reassessment if clinical signs have resolved.

The six cases with suspected or confirmed orbital lesions in this study are notable. These cases could be presumed to be suffering from invasive fungal infections, but 3/3 cases with positive fungal cultures were diagnosed with *Aspergillus fumigatus* on culture alone, which is usually non‐invasive and associated with sino‐nasal rather than sino‐orbital disease [17, 19]. This might represent misidentification of the fungal isolates [17], and unfortunately, the retrospective nature of this study precluded our ability to perform molecular identification methods on these isolates. Interestingly, the rates of long‐term remission (> 90‐days) were not dissimilar for orbital involvement, with 2/5 cases achieving long‐term remission. However, it should be noted that these results are from a small number of cases and the caveats of identifying remission discussed should be considered.

Additional inherent limitations of this study paper relate to its multicentre, retrospective nature. There were no defined search criteria to identify cases due to the heterogeneous nature of practice management systems across centres, and this combined with the inherent bias introduced by enrolling cases purely from referral centres precluded assessment of disease prevalence. Each investigator aimed to implement multifaceted search methodologies to identify all possible cases, but the possibility of bias resulting from a non‐standardized approach cannot be fully excluded. Diagnostic investigations and treatment recommendations were not standardized and were clinician‐dependent. In addition, the frequency of some findings might be underestimated due to incomplete medical records. Variability in clinician experience and their interpretation of test results might have impacted the diagnostic performance of certain diagnostics such as endoscopy and the acquisition of endoscopic‐guided biopsies. CT images were not available for review, so the description of disease extent (including orbital involvement) was based on historical imaging reports. As previously discussed, despite the inclusion of multiple referral centres, due to the rarity of FRS in cats, the small number of cases and heterogeneity of this population makes firm conclusions, particularly relating to treatment efficacy and prognosis, difficult to draw. Furthermore, cats with various fungal aetiologies (as well as those without fungal speciation) were included, and it is possible that the disease courses, optimal treatment, and outcomes might differ for each. Further prospective studies are warranted to further investigate these factors and evaluate the disease prevalence in both primary care and referral veterinary practice.

In conclusion, this study provides insight into the presentation, investigations, treatment, and outcome for FRS in cats in the United Kingdom. Fungal rhinosinusitis should be considered in any cat presenting with chronic nasal disease regardless of age and breed, and multifaceted investigations, including advanced imaging, rhinoscopy, histopathology, fungal culture, and panfungal PCR, should be performed to confirm or exclude this diagnosis. Both topical and systemic antifungal therapy were well tolerated in this study with a low rate of adverse effects. Finally, whilst FRS was an uncommon reason for death or euthanasia in this study, the prognosis for cats with this condition appears to be guarded, with > 50% of cases suffering from persistent or relapsing clinical signs.

## Disclosure

Off‐label use of antimicrobials prescribed according to individual clinician judgment and recommendation included: systemic posaconazole, systemic fluconazole, topical clotrimazole 1% cream, and topical clotrimazole 1% solution.

## Ethics Statement

Approval granted by the ethics and welfare committee at the Department of Veterinary Medicine, University of Cambridge (C643). Authors declare human ethics approval was not needed.

## Conflicts of Interest

The authors declare no conflicts of interest.
